# Fibre: The Forgotten Carbohydrate in Sports Nutrition Recommendations

**DOI:** 10.1007/s40279-024-02167-1

**Published:** 2025-01-08

**Authors:** Laura Mancin, Louise M. Burke, Ian Rollo

**Affiliations:** 1 Research Institute for Sport and Exercise Sciences, Liverpool John Moores University, Liverpool, UK; 2 Mary MacKillop Institute for Health Research, Australian Catholic University, Melbourne, VIC Australia; 3 Gatorade Sports Science Institute, PepsiCo Life Sciences, Global R&D, Leicester, UK; 4 School of Sports Exercise and Health Sciences, Loughborough University, Loughborough, UK

## Abstract

Although dietary guidelines concerning carbohydrate intake for athletes are well established, these do not include recommendations for daily fibre intake. However, there are many scenarios in sports nutrition in which common practice involves the manipulation of fibre intake to address gastrointestinal comfort around exercise, or acute or chronic goals around the management of body mass or composition. The effect of fibre intake in overall health is also important, particularly in combination with other dietary considerations such as the elevated protein requirements in this population. An athlete’s habitual intake of dietary fibre should be assessed. If less than 20 g a day, athletes may consider dietary interventions to gradually increase intake. It is proposed that a ramp phase is adopted to gradually increase fibre ingestion to ~ 30 g of fibre a day (which includes ~ 2 g of beta-glucan) over a duration of 6 weeks. The outcomes of achieving a daily fibre intake are to help preserve athlete gut microbiome diversity and stability, intestinal barrier function as well as the downstream effects of short-chain fatty acids produced following the fermentation of microbiome accessible carbohydrates. Nevertheless, there are scenarios in which daily manipulation of fibre intake, either to reduce or increase intake, may be valuable in assisting the athlete to maintain gastrointestinal comfort during exercise or to contribute to body mass/composition goals. Although further research is required, the aim of this current opinion paper is to ensure that fibre is not forgotten as a nutrient in the athlete’s diet.

## Key Points

**Table:** 

Modifying the fibre content of the diet is a common sports nutrition strategy used by athletes, based on the clinical judgement and expertise of sports nutrition practitioners.
There is a lack of randomly controlled clinical trials to support fibre intake recommendations in athletes.
Maintaining daily fibre intake is likely to support athletes’ microbiome stability and gastrointestinal function.

## Introduction

Dietary guidelines concerning carbohydrate intake for athletes are well established [1–3]. Daily carbohydrate consumption is recommended to be adjusted depending on the demands of exercise, with a range of 3–5 g of carbohydrate per kilogram of an athlete’s body weight for “light” activity, to 8–12 g per kilogram of body mass for “very high” activity [4]. These targets are intended to ensure sufficient carbohydrate availability for the muscles and central nervous system in response to the respective exercise demands. The timing of carbohydrate ingestion is also adjusted to ensure carbohydrate availability before or during a specific competitive event or training session, as well as during recovery from prolonged or intensive exercise [1, 5].

Athletes are encouraged to choose nutrient-rich carbohydrate foods to meet daily targets [4]. However, this can be challenging when aiming to consume high quantities of carbohydrate. In these circumstances, athletes ingest energy-dense carbohydrate-rich foods that often contain less nutrients and of particular interest, fibre. Fibre ingestion is recommended for the general population owing to its association with numerous health benefits including a reduced risk of cardiovascular disease [6] and the lowering of blood low-density lipoprotein-cholesterol [7]. In addition to these benefits, which could potentially extend to athletic populations, there is evidence that dietary fibre offers a direct benefit to gastrointestinal function in healthy individuals [8–11]. Specifically, fibre may modulate health through the interaction with the gut microbiome [12].

Therefore, it is surprising that the only “official” fibre-related recommendations in the American College of Sports Medicine sports nutrition position statements for athletic performance are concerned with limiting fibre intake prior to exercise. Here, athletes are advised to choose easy-to-consume carbohydrate-rich sources that are low in fibre/residue to reduce the risk of gastrointestinal complaint during exercise [4]. However, practicising sports dietitians often recommend/implement strategies that manipulate the fibre content of the athlete’s diet to achieve acute or individualised goals. These strategies are often based on clinical expertise, anecdotal experiences or the re-application of practices developed in clinical nutrition scenarios (e.g. weight management, gastrointestinal issues). Table 1 summarises common scenarios where dietary fibre is manipulated in sport, noting that the evidence base that supports their use, notwithstanding some studies of specific applications that are noted in the table, could mostly be considered Level 7 (based on clinical expertise and personal experience of nutrition experts).

**Table 1:** Common scenarios where dietary fibre intake is modified in sport

Scenario	Sample athlete	Advice or practices with fibre intake	Comments
**Default:** Prevention/reduction of GI symptoms during exercise (exercise-associated GI symptoms)	Individual athletes with a high risk of GI symptoms during exercise or exercise scenarios of high risk (prolonged/high intensity), hot weather	Reduce fibre in pre-exercise/event meal(s)	General advice to reduce the risk or actual episodes of general GI discomfort during exercise based on anecdotal reports and self-reported practices [15, 16] Included in specific management of ex-GI symptoms in athletes with more pronounced problems [17] based on clinical experience
		FODMAP management strategy: 24–48 h low intake of foods with high FODMAP content (fermentable oligo-, di-, and mono-saccharide and polyols)	Included in specific management of ex-GI symptoms in athletes with more pronounced problems [17] based on clinical experience and results of intervention studies [18, 19]
**Performance:** Carbohydrate-loading diet (maximisation of pre-event glycogen storage for endurance events)	Marathon runner Ironman triathlete	48–72 h of high carbohydrate intake (10–12 g/kg/day) based on a low- residue/fibre diet (see Table 2). Switching to a low-residue/fibre diet for 48–72 h can reduce the bulk of GI contents to offset the associated increase in BM, reduce GI discomfort from increased food intake and reduce the need for a bowel movement on race day	Carbohydrate-loading practices are well supported as preparation for endurance events [20, 21]. Integration of a low-residue/fibre diet with the carbohydrate-loading plan, based on clinical experiences with bowel prepation for GI surgery is intuitive, in terms of practical benefits of reducing the mass and toilet requirements of normal bowel contents. It has become a common recommendation within carbohydrate-loading protocols [21] (Table 3) based on clinical experiences [20] and testimonials from athletes [22]
**Performance:** High-energy needs (energy requirements exceed GI comfort associated with food intake)	Tour de France cyclist Heavy weight rower Adolescent male swimmer	Reduce fibre-rich foods and favour energy-dense compact foods and drinks (usually fibre poor) within the meal plan	Intuitive advice to increase the energy density of the diet and food choices to allow daily intakes > 6000 kcal to be achieved within GI comfort and available time for food intake. Reflects sample food plans written for athletes or food practices reported by athletes [23, 24]. Relative displacement of fibre-rich foods and absolute fibre intake may still vary between and within athletes
**Performance:** Carbohydrate intake during endurance exercise to supply additional muscle fuel source	Marathon and ultra-marathon runner, runner Road cyclist Cross country skier Triathlete (especially Ironman)	Consume 30–90 g/hour of carbohydrate according to the duration and intensity of the exercise session. When consuming > 60 g/hour, make use of “multiple transportable carbohydrate” sources—products containing glucose-based carbohydrates and fructose—to take advantage of greater intestinal absorption from different intestinal transport mechanisms. Choose sources that are easy to consume and tolerate during exercise, especially those that are low in fibre	Use of low-fibre carbohydrate-rich foods and fluids is intuitive to reduce risk of ex-GI symptoms and to promote ease of consumption of higher targets. Specialised sports foods/drinks and everyday processed foods may assist with practical aspects of access and consumption during exercise, as well as provide augmented composition (e.g. multiple transportable carbohydrate or addition of caffeine and other performance aids [25, 26]). In competition scenarios, ultra-processed foods/sports foods may be preferred for GI comfort or represent the typical options provided for nutrition support. Anecdotal reports [27] and observational studies [24, 28, 29] support such practices in athletes
**Performance:** Suppressed appetite post-training or competition impedes recovery nutrition goals	Heavyweight rower with two to three times a day training Swimmer with twice-daily training sessions Tour de France cyclist	Promote energy-dense compact forms of protein or carbohydrate-rich recovery foods (usually fibre poor) to overcome loss of appetite or other practical considerations of post-exercise scenarios (e.g. processed foods that can be easily stored or prepared)	Athletes who undertake high-intensity training sessions, especially in hot conditions, often report suppressed appetite at the end of each session. During periods of high-volume training or strenuous competition, post-exercise intake contributes to faster recovery as well as total daily energy intake. It is intuitive to choose processed foods and liquid forms of carbohydrate and protein, including specialised sports foods, to enable consumption when appetite or reduced access to food storage/preparation limits other choices [25, 26]
**Targeted:** Weight management (energy restriction to create an energy deficit or meet low-energy requirements in athletes needing to reduce BM or body fat)	Athletes in “aesthetic” sports with lower energy requirements (e.g. gymnastics, diving) Athletes actively undertaking loss of BM or body fat	Increase intake of fibre-rich foods and promote lower energy density (often fibre rich) to increase meal volume and satiety	Intuitive advice to reduce the energy density of the diet and food choices to allow reduced energy intakes to be achieved while maintaining a high volume of food. Reflects sample food plans written for athletes or food practices reported by athletes during periods of reduced energy intake [30, 31]
**Targeted:** Weight making (contribute to acute BM reduction to meet weigh-in target)	Combat sports Lightweight rowing Weightlifting	48–72 h of low-residue/fibre diet to contribute the loss of bowel contents to overall BM loss	Athletes in weight division sports often resort to severe energy restriction in the pre-competition period to reduce BM. The integration of a low-residue diet with other weight-loss strategies is intuitive in reducing the mass of bowel contents while still enabling energy/macronutrient intake for competition fueling. This strategy is integrated into official [32] and general [32] recommendations for safer weight making. One study of weight making in combat athletes has reported a substantial BM reduction (1.5% BM), attributed to the low-fibre diet within an overall strategy [33]

The default scenario is paired with menus provided in Menu 1, performance-orientated scenarios are paired with Menu 2 and targeted scenarios are paired with Menu 3, displayed in Table 3

*BM* body mass, *FODMAP* Fermentable Oligosaccharides, Disaccharides and Monosaccharides and Polyols

**Table 2:** History of dietary fibre classification, with corresponding foods

Definition of dietary fibre content	Basis	Example of foods considered “high” vs “low” fibre
High vs low fibre (1950–80 s) [45–46]	No differentiation of different types of fibre Dietary fibres generally considered as the intrinsic non-digestible constituents that are resistant to hydrolysis by human enzymes, which included cellulose, hemicellulose, lignin and associated minor substances such as waxes, cutin and suberin	High fibre: wholegrain cereals, whole fruits and vegetables (especially unpeeled and uncooked) Low fibre: “white” cereals, white rice and pasta, baked mashed potatoes, fruit juice, cornflakes, cooked vegetables, fruits without skin
Soluble vs insoluble fibre (2002) [46]	The ability of a fibre to dissolve in water Water-soluble fibre/well-fermented fibres are water soluble and derived from the inner flesh of plants such as pectin, gums and mucilage. Form a viscous gel and are usually fermented by bacteria in the colon. They produces gases and by-products such as short-chain fatty acids Water-insoluble/less-fermented fibres derive from the outer skin of plants. Insoluble in water and cannot undergo fermentation by bacteria in the colon. As a result, they produce less gases, form the bulk of the stool and promote laxation Cellulose, hemicellulose, and lignin are insoluble fibre	High-soluble fibre: whole fruits, barley, avocado, oatbran, beans, lentils, sweet potatoes, chia seeds, oats, psyllium husk Low-soluble fibre: flax and sesame seeds, peanuts dry roasted, coconut raw, almonds, kiwifruit, pineapple, unpeeled apple, strawberry, raw green vegetables, eggplant, potatoes with skin
Fermentable vs non-fermentable fibre (1990) [47, 48]	Fermentable fibres are readily metabolised by the gut microbiota. Fermentation of fibre results in the formation of microbially derived compounds such as short-chain fatty acids (acetate, propionate and butyrate) and gases (H_2_, CO_2_) Non-fermentable/bulking fibres create larger softer stools and speed the passage of waste through the digestive tract. These types of fibres pass through the intestine relatively intact and are useful for the prevention or treatment of constipation	High fermentable fibre: oats, barley, onion, artichokes, garlic, legumes, pulses (canned chickpeas and lentils), asparagus, psyllium husks, cabbage, guar-gum and xanthan gum supplements Low/non-fermentable fibre: whole wheat, whole grain, wheat bran, green beans, potatoes, flax and sesame seeds, rye, mature root vegetables, broccoli (stalk), fruits with edible seeds such as berries, apricots, grapes, almonds and walnuts
Microbiota-assessible carbohydrates (2014) [10]	The concept of dietary MACs is essentially equivalent to that of fermentable dietary fibres with the additional value of the individuality of the gut microbiota in their capacity to utilise certain fermentable dietary fibres	The amount of dietary MACs present in a single food source differs between individuals Dietary fibre metabolism depends on individual microbiota composition. For example, lactose becomes a substrate for the microbiota in people who are lactose intolerant and therefore it represents a MAC only for these individuals

*MAC* microbiota-accessible carbohydrate

**Table 3:** Nutrient profiles of 3-day menus adjusting the fibre content to different athletes’ diet scenarios (Table 1)

***Menu 1: default scenario: high fuel, high fibre***	
**Day 1**	
**Total fibre: 33.8 g** 14.2 MJ (3390 kcal) 550 g carbohydrate (10 g/kg BM) 108 g protein (2.0 g/kg BM) Note that this menu represents health-focused or energy-balanced nutrition choices Advice remains to avoid high-fibre food choices in the hours prior to training to reduce the risk of a gastrointestinal complaint during exercise The lunch options are deliberately lower in fibre because afternoon training sessions typically involve high-intensity running, which increases the risk of a gastrointestinal upset Snacks consumed during and after training sessions are low fibre to reduce gut discomfort and encourage intake despite a dampened appetite after high-intensity workouts	**Breakfast** 4 pancakes 3 tbsp maple syrup 1 cup blueberries 250 mL fresh apple juice
	**Lunch** 300 g pizza margherita 2 cups Jello
	**Dinner** 250 g cooked whole wheat pasta 1 cup tomato puree 150 g cooked chicken breast 1 cup cooked broccoli 2 cups cooked zucchini 1 tbsp extra virgin olive oil 250 mL fresh apple juice
	**Snacks over the day** 1000 mL sports drink 170 g flavoured yoghurt
**Day 2**	
**Total fibre: 33.9 g** 13.7 MJ (3270 kcal) 550 g carbohydrate (10 g/kg BM) 95 g protein (1.7 g/kg BM)	**Breakfast** 200 g yoghurt 90 g granola 1 tbsp honey 1 medium banana 1 tbsp chia seeds 200 mL fresh orange juice
	**Lunch** 2 cooked lean beef patties (150 g) 300 g baked potato 6 asparagus 1 cup cooked carrots 2 tbsp honey mustard sauce
	**Dinner** 2 burritos (with chicken, corn, rice, mixed vegetables) 100 g ice cream
	**Snacks over the day** 2 bottles of sports drink 1 medium apple 200 g rice pudding
**Day 3**	
**Total fibre: 36 g** 13.2 MJ (3150 kcal) 550 g carbohydrate (10 g/kg BM) 139 g protein (2.5 g/kg BM)	**Breakfast** Porridge with 70 g oats, 250 mL milk 2%, 1 tbsp honey, 30 g dark chocolate (80% cacao)
	**Lunch** Sandwich (150 g baguette, omelette with 2 eggs and 1 egg white, 2 slices light cheese, 1 slice ham) 1 cup grilled vegetables 250 mL smoothie (fruits and milk)
	**Dinner** Pasta salad (280 g cooked penne, 120 g tuna canned in water, 200 g mixed vegetables) 250 g Jello
***Menu 2: performance scenario, high fuel, low fibre***	
**Day 1**	
**Total fibre: 18.8 g** 14.2 MJ (3390 kcal) 550 g carbohydrate (10 g/kg BM) 108 g protein (2.0 g/kg BM) Note that many athletes may not choose to undertake this low-residue (low-fibre) style of carbohydrate-loading menu. In this case, fruits and vegetables can be integrated into the meals. In addition, the athlete may choose wholegrain versions of the cereal foods included in the menu. Furthermore, a loading period of 48 h may be sufficient for some athletes or races This menu does not represent health-focussed or energy-balanced nutrition choices. Rather, it targets low-fibre food choices and sugar-rich foods to deliberately meet carbohydrate goals for maximal glycogen synthesis, from a manageable volume of food. Jelly represents a food low in fibre but provides a good source of carbohydrate and fluid This is a specialised nutrition strategy to meet the needs of an occasional event, for example a marathon for which the athlete is targeting peak performance	**Breakfast** 1.5 cups rice Krispies cereal 200 mL milk
	**Lunch** 100 g white focaccia roll with ham and cheese filling 170 carton flavoured yoghurt (no added fruit pieces) 250 mL pulp-free orange juice
	**Dinner** 2.5 cups cooked white pasta spirals 1 cup bolognaise sauce made with lean minced meat and tomato puree (no vegetable pieces) 200 g (3 medium scoops) ice cream + 60 mL flavoured topping
	**Snacks over the day** 600 mL sports drink 120 g jelly confectionary
**Day 2**	
**Total fibre: 16.9 g** 13.7 MJ (3270 kcal) 553 g carbohydrate (10 g/kg BM) 95 g protein (1.7 g/kg BM)	**Breakfast** 3 cups corn flakes 300 mL milk 250 mL pulp-free apple juice
	**Lunch** 1 large (180 g) California roll sushi (remove nori wrapping) 300 mL flavoured reduced fat milk 2 × Rice Krispie squares
	**Dinner** Half large margherita pizza (250 g) 250 mL pulp-free apple juice 2 toasted waffles (90 g each) 40 g maple syrup 2 small scoops ice cream (90 g), small bowl of ice cream (90 g)
	**Snacks over the day** 1200 mL sports drink 2 × 150 g cartons of creamed rice/rice pudding
**Day 3**	
**Total fibre: 10.8 g** 13.2 MJ (3150 kcal) 550 g carbohydrate (10 g/kg BM) 139 g protein (2.5 g/kg BM)	**Breakfast** Stack of 4 medium pancakes (240 g) 60 g golden syrup 150 g tub vanilla sweetened yoghurt 250 mL pulp-free orange juice
	**Lunch** Half baguette (130 g) Chicken breast filling (150 g) 250 mL pulp-free orange juice
	**Dinner** 2 cups cooked white rice 2 lean beef kebabs (150 g total) 80 g honey soy sauce 1 cup Jello 1 tub (100 g) dairy dessert/mousse
	**Snacks over the day** 1200 mL sports drink 150 g flavoured yoghurt 100 g jelly confectionary
***Menu 3: targeted scenario, low fuel, high fibre***	
**Day 1**	
**Total fibre: 27 g** 8 MJ (1900 kcal) 165 g carbohydrate (3 g/kg BM) 137.5 g protein (2.5 g/kg BM) Note that this menu represents health-focused dietary choices for an energy restriction to create an energy deficit or meet low-energy requirements in athletes needing to manage BM or body fat Athletes in weight division sports often combine an energy restriction with low-residue foods as an acute BM reduction strategy. In these situations, wholegrain versions of the foods included in the menu can be replaced with low-fibre alternatives At competition times, the diet could be modified to reduce fibre (to assist with weight loss/body aesthetics) by changing to white cereals, removing vegetables/fruit in favour of small amounts of purees or clear juices	**Breakfast** 50 g oats 250 mL milk 2% 1 tsp honey 1 medium apple with skin
	**Lunch** Sandwich (150 g whole wheat bread, 2 medium slices ham, 2 slices light cheese, tomato and cucumber) 150 g Greek yoghurt 1 tbsp peanut butter 10 almonds
	**Dinner** 150 g whole wheat pasta cooked 1 cup bolognaise sauce made with lean minced meat and tomato puree (with vegetable pieces) 1 cup mixed vegetables
	**Snacks over the day** 1 protein pudding (200 g) 1 medium banana
**Day 2**	
**Total fibre: 25 g** 8 MJ (1900 kcal) 165 g carbohydrate (3 g/kg BM) 137.5 g protein (2.5 g/kg/BM)	**Breakfast** 20 g all-bran buds 200 g Greek yoghurt 1 tsp honey 2 kiwifruit
	**Lunch** 160 g cooked salmon 1 cup cooked brown rice 1 cup mixed vegetables
	**Dinner** 2 slices whole wheat bread 1 slice turkey breast 2 slices light cheese 2 boiled eggs 1 crepe with 1 tbsp peanut butter
	**Snacks over the day** 1 protein pudding (200 g) with 1 cup berries 1 medium banana with 1 tbsp almond butter
**Day 3**	
**Total fibre: 29 g** 8 MJ (1900 kcal) 165 g carbohydrate (3 g/kg BM) 137.5 g protein (2.5 g/kg BM)	**Breakfast** 2 slices whole wheat bread ½ avocado 2 boiled eggs 2 medium slices cheese Small glass of fresh orange juice
	**Lunch** 4 medium falafels 2 slices white pitta bread Small side salad 100 g Greek yoghurt (dip)
	**Dinner** 200 g cooked quinoa 150 g cooked chicken breast 2 cup cooked mixed vegetables
	**Snacks over the day** 100 g Greek yoghurt with 1 cup berries

Example based on a 55-kg athlete (runner). Menu 1 provides foods to achieve a high-carbohydrate high-fibre healthy/long-term diet during intensive training. This diet may be considered the default scenario, with a reduction in fibre around training sessions to aid energy intake and reduce gastrointestinal disturbances. Menu 2 provides foods to achieve a low-residue high-carbohydrate intake to maximise pre-event glycogen storage for endurance events. This diet may be considered for performance-orientated scenarios. Menu 3 provides foods for targeted scenarios aimed to achieve a low-carbohydrate intake for low fueling for weight management and energy restriction days but to maintain an adequate fibre intake. Drinking 5–7 mL of water per kg BM with the first meal of the day, after which fluid intake should be modified to individual requirements. All menus analysed using Nutritium Ltd. (https://nutrium.com/en)

*BM* body mass

Dietary carbohydrates, including simple sugars and starches, represent the largest fraction in sport nutrition recommendations. However, total carbohydrates in most foods are currently quantified indirectly by a gravimetric mass method, resulting in a nutritional “blind spot” [13]. Correspondingly, despite being an abundant component of foods, the structure, quantity and function of fibre is also poorly characterised [14]. Current approaches broadly categorise dietary carbohydrate into sugars, starch and soluble/insoluble fibre, which provides inadequate information on specific chemical or structural content [14]. Thus, we recognise the need to understand the influence of different physicochemical characteristics of dietary fibres to inform fibre-specific strategies for an athlete’s daily health and competitive performance.

To inform the present article, an electronic literature search was undertaken using four online databases (PubMed, Web of Science, MEDLINE, Google Scholar). Searches were performed using keywords from existing relevant papers. Search terms were “fibre/fiber”, “exercise”, “carbohydrate”, “health” and “performance” phrased as appropriate. Reference lists of all studies and relevant systematic reviews were examined manually to identify relevant studies for this review.

To our knowledge, there is a lack of consolidated expert guidance for athletes on daily dietary fibre ingestion, especially advice that can integrate total nutrient and health-related requirements within sports nutrition-specific strategies. Thus, the aim of the current opinion paper was to discuss dietary fibre as an important consideration to support an athlete’s gastrointestinal health, while providing guidance concerning acute ingestion around exercise and noting other sports-specific issues.

## Dietary Fibre Classification

**Fig. 1:**
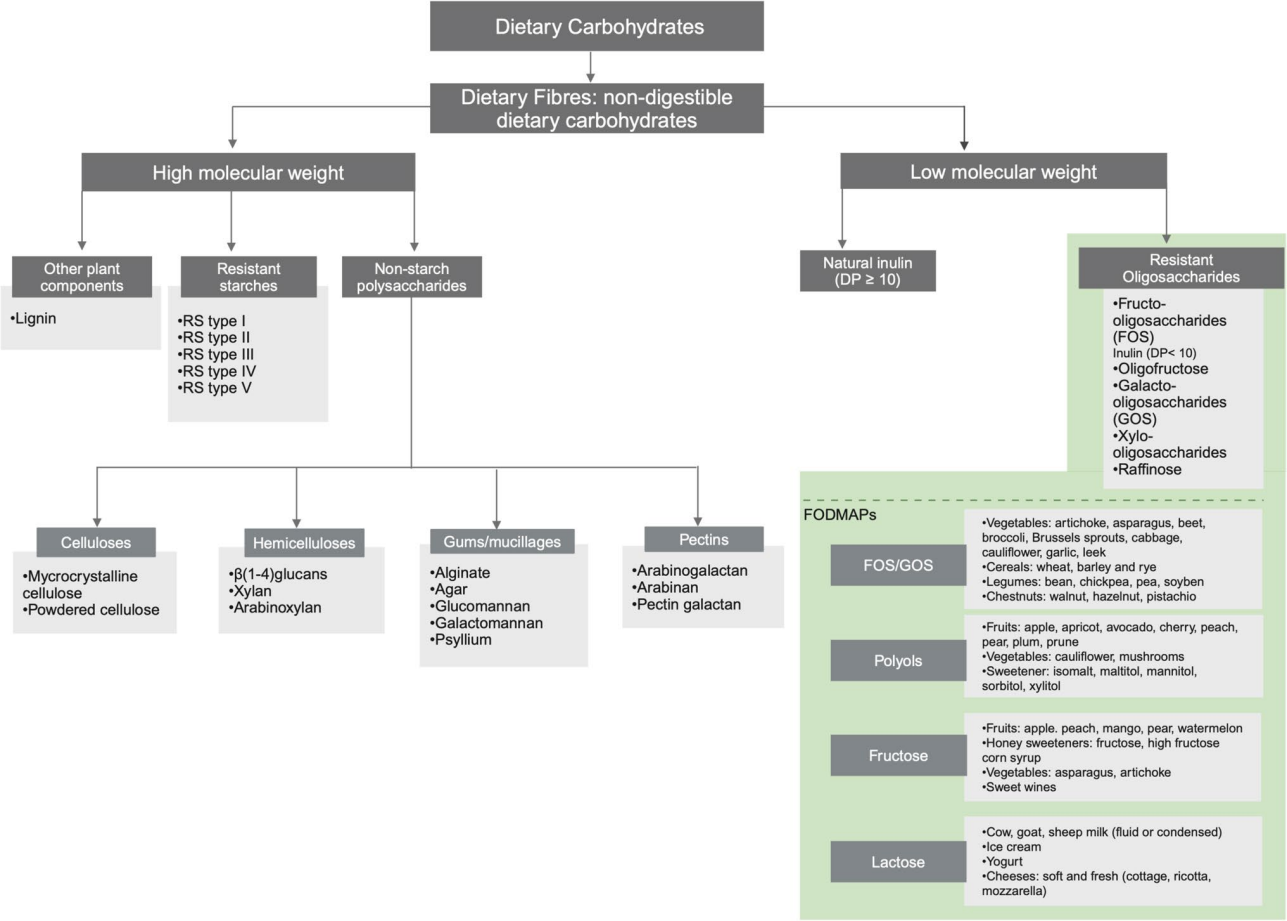
Dietary fibre classification based on molecular weight. Categories of the different types of non-digestible carbohydrates according to molecular weight. Non-starch polysaccharides, resistant starches (RS) [types 1–5] and lignin are high-molecular-weight dietary fibres. Resistant oligosaccharides (including short-chain inulin degree of polymerisation [DP] ≤ 10, as fructooligosaccharides and oligofructose) and natural inulin (DP value in range 2–60) are low molecular weight. Fructooligosaccharides and galactooligosaccharides are considered as well as Fermentable Oligosaccharides, Disaccharides and Monosaccharides and Polyols **(**FODMAP) compounds and included within the FODMAP group

Dietary fibres are classified according to their physicochemical structures [8, EC. Regulation (EU) No. 1169/201135]. Historically, dietary fibre has been classified as soluble and insoluble. Insoluble-non-viscous fibre remains unchanged during food transit in the body. As an indigestible material, insoluble fibre sits within the gastrointestinal tract, absorbing fluid and sticking to other by-products of digestion that are ready to be formed into the stool. Soluble-viscous fibre (i.e. psyllium) dissolves in water and gastrointestinal fluids when it enters the stomach and intestines. The fibre is modified into a gel-like substance, which is available for bacteria digestion in the large intestine [8, 35–37]. However, this classification is limited owing to a number of reasons. For example, both psyllium (soluble) and cellulose (insoluble) improve glycaemic control and transit time although through different mechanisms: increasing the viscosity of intestinal content and the inhibition of starch digestibility, respectively. However, solubility has a strong effect on the other aspects such as viscosity and fermentability. Indeed, dietary fibre can also be classified based on viscosity. Viscosity, which is the ability of a fibre to hold water and thicken when hydrated, is generally associated with soluble dietary fibres such as beta-glucans and psyllium. Increasing luminal viscosity through viscous dietary fibres (turns to gel during digestion) has been suggested to have many physiological effects such as decreasing the gastric emptying rate, modulating the small intestine transit, softening hard stool in constipation and normalising stool form [38, 39]. Correspondingly, dietary fibres can be categorised as fermentable or non/low fermentable. In this classification, fermentable carbohydrate also includes those short-chain sugars that are easy to break down and are available for gut bacteria to ferment. Whereas, non-fermentable sugars are not available to the gut bacteria for fermentation [8, 36] (Table 2). The detailed functional properties and physicochemical characteristics of different dietary fibres have been extensively reviewed by So et al. and Gill et al. [40, 41] (Fig. 1).

In line with this concept, in the last decade, the concept of “Fermentable Oligosaccharides, Disaccharides and Monosaccharides and Polyols, FODMAP” has been introduced to describe certain fermentable foods that are able to trigger gastrointestinal symptoms (e.g. gas, diarrhoea and abdominal bloating) in individuals with irritable bowel syndrome [42, 43]. FODMAP foods have low digestibility in the upper gastrointestinal tract and rapid bacterial fermentation in the proximal colon. Foods high in FODMAPs, given their small size and high osmotic activity, foster water mobilisation in the intestine, cause abdominal distention and increase gas production [43]. Although the induction of symptoms related to FODMAP is not fully understood, a low FODMAP dietary approach has been associated with the onset of gastrointestinal symptoms in endurance-type athletes [17, 18]. It is worth noting that dietary fibres, including non-digestible oligosaccharides (galactooligosaccharides) and inulin (fructans), are also the main components to be excluded of the low FODMAP approach, as they are the most abundant dietary fibres found in wheat products, pasta, breads and breakfast cereals.

The definition of dietary fibre continues to evolve [34]. Attempts to use soluble versus insoluble fibre as conduits of fermentable versus nonfermentable fibre, respectively, have proven imprecise [9]. In response, Sonnenburg and Sonnenburg [10] introduced the concept of classifying dietary fibres based on their availability as a substrate for the microbiome. These ‘microbiota-accessible carbohydrate’’ (MACs) [10] are defined as carbohydrates that are metabolically available to gut microbes. The MACs can be divided into two categories, dietary and host derived. The dietary MACs are resistant to degradation and absorption whereas host-derived MACs are secreted by the host or produced by microbes within the intestine [10] (Table 2).

Nonetheless, determining whether a dietary carbohydrate is a MAC is complicated by the individual response to its utilisation. The enzymatic capacity of an individual microbiota will determine the ability to digest the carbohydrate. As an example, resistant starch (RS) type 3 is typically considered a MAC, as it is metabolised by human microbiota [8]. However, if a microbiome lacks the keystone species *Ruminococcus bromii,* the carbohydrate will not be metabolised [49]. Similarly, beta-fructan fibres may be beneficial in individuals with a high fermentative potential but potentially proinflamatory in individuals lacking fermentative microbes [50]. Further complexity is added by the host genotype, which can determine the quantity of dietary MACs derived in a single food source. A common example is lactose, which becomes a substrate for the microbiota in individuals with lactose intolerance, and thus in this instance should be classified a MAC [51, 52].

**Fig. 2:**
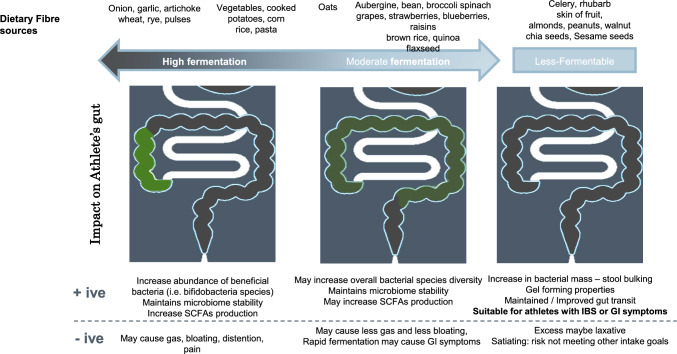
Fermentation capacity of dietary fibre and associated positive (+ ive) and negative (− ive) impact of the athlete’s gut. *GI* gastrointestinal, *IBS* irritable bowel syndrome, *SCFAs* short-chain fatty acids

Despite complexities, MACs can be generally be compared to that of fermentable dietary fibre, with the additional criterion regarding the individuality of the gut microbiota composition and its fermentative potential. Therefore, when discussing the impact of dietary fibre on the gut microbial community (intestinal microbiota composition and function and intestinal barrier [through short chain fatty acid-dependent mechanisms]), dietary fibre is best classified as non-fermentable or MACs (Fig. 2).

## Dietary Fibre (MACs) and Gut Microbiota Composition

The ingestion of MACs in the diet of healthy adults significantly alters the microbial community by providing the nutritional substrates for microbial growth. The ingestion of RS has been shown to increase the abundance of bacterial commensals such as *Bifidobacterium adolescentis* and *Ruminococcus bromii* [53]. The ingestion of MACs also increases the production of fermentation end products, which in turn influence host physiological processes [10]. Interestingly, the enrichment of bacterial species appears dependent pn the different types of RS ingested (RS type 2 vs type 3 vs type 4) [49, 54], indicating that changes in the microbial community are associated with the chemical structure of the fibre. It is important to note that the response of a specific bacterial group to a dietary MACs is not only determined by the capacity to adhere to the substrate but also the ability to tolerate new environmental conditions generated from the fermentation (i.e. low pH). Although the microbial response and growth may be fibre dependent, the available evidence demonstrates that non-fermentable carbohydrate ingestion leads to increased microbial diversity when compared with a fibre-deprived diet [55]. Although “optimal” microbiome profiles are yet to be established, it is generally accepted that diverse and stable microbiomes are superior to more homogenous profiles in respect supporting gut health [9, 56].

## Gut Function and Dietary Fibre Microbiota Interactions

Dietary fibres that escape digestion by host enzymes in the upper gut have the ability to change the profile of microbially derived metabolites in the cecum and colon. The fermentation of MACs results in the production of beneficial end products such as SCFAs. The SCFAs are organic products such as acetate, propionate and butyrate, which possess regulating capacities on whole body systems including immune function [57, 58], neurological processes [59] and skeletal muscle metabolism [60].

Of the SCFAs, butyrate has been investigated the most extensively. Upon absorption, butyrate is metabolised by the intestinal epithelial cells (colonocytes) serving as a major energy source. Several studies have reported that a low intake of MACs resulted in reduced microbial diversity and reduced SCFA production. Conversely, a high-dietary fibre intake enhanced SCFA production, which in turn maintained epithelial integrity and strengthened intestinal barrier function [61, 62]. The mechanism by which butyrate enhances intestinal barrier function is through the upregulation of genes encoding tight-junction proteins (claudin-1 and Zonulin-1) [64–66], whilst simultaneously reducing the concentration of lipopolysaccharides and systemic inflammation [67].

In diets deprived of MACs, gut microbial metabolism can shift towards the utilisation of dietary and endogenous protein, which may have a negative impact on gut function [68, 69]. Adults supplied with a high-protein and low-fermentable carbohydrate diet reduced the production of SCFAs as well as increased less favorable metabolites derived from the fermentation of amino acids, including ammonia, amines, *N*-nitroso compounds, phenolic compounds and *p*-Cresol [70]. A low intake of fermentable dietary fibres also shifts the microbial metabolism towards the utilisation of host-derived substrates such as mucosal glycoproteins. This response can lead to a depletion of the mucus layer increasing intestinal permeability, gastrointestinal inflammation and susceptibility to pathogen invasion [71, 72].

## The Athletic Diet

As summarised in the introduction, the known sports nutrition strategies that manipulate fibre/“residue” over acute or chronic periods are commonly used by, or recommended to athletes, based on clinical judgement and experience (Table 1). However, it is unknown how these strategies or indeed the general nutrition practices of athletes affects total fibre content and type. Consistent with the general population, the only available data [74–75] suggest that the daily consumption of fibre by some athletes fails to meet the recommendations for health in the general population (25 g for female individuals and 38 g for male individuals) [76]. For instance, self-reported intake of fibre in groups of endurance-trained athletes was less than 25 g per day [73], whereas the fibre ingestion in high-level soccer players, body builders and distance runners was assessed at approximately 16, 19 and 17 g per day, respectively [74, 77]. Of course, these assessments are limited by the constraints of dietary survey methods, which are likely to underestimate the true energy intake in athletes by ~ 20% [78]. Additional constraints include different definitions of dietary fibre and general inadequacy of the food composition data in the reference databases used to undertake these surveys. Guidelines of fibre intake are required as too much fibre is likely to delay digestion and absorption as well as cause bloating, gas production, nausea and weight changes [79, 80], whereas too little would result in constipation, gut barrier impairments and less favourable gut microbiota [81].

## Athlete Dietary Practices and the Microbiome

There is little systematic study of the effect of fibre intake, or other non-fibre characteristics of the athlete’s diet on the gut microbiome. In terms of manipulations to carbohydrate and fibre intake, one study reported little change in characteristics of the microbiome (pH and abundance of *Faecalibacterium prausnitzii, Akkermansia muciniphila, Bifidobacterium* spp. and *Bacteroides spp* bacteria) when amateur cyclists increased their cycling volume and their intake of non-fibre-containing carbohydrate-rich foods during their sports season [82]. Meanwhile, a 3-week exposure to a ketogenic low-carbohydrate high-fat diet in elite race walkers, altering the type and amount of fibre intake, resulted in a change in enterotypes with a greater relative abundance of *Bacteroides* and *Dorea* and a reduction in *Faecalibacterium* [83]. Concurrently, sports nutrition guidance recommends elevated dietary protein ingestion, primarily to support skeletal muscle adaptation following exercise [84]. This is relevant as an elevated protein intake can result in a reduced gut microbial diversity, when athletes follow a low-carbohydrate diet or eat low-fibre foods [77].

Maintaining a sufficient dietary intake of MACs appears to protect microbiome diversity in response to increasing dietary protein ingestion. When professional rugby players achieved the recommended intake of carbohydrate and fibre, as the protein intake increased, the gut microbiota diversity also increased [85]. Furthermore, when diets high in protein or fat intake are supplemented to include dietary fibre, beneficial microbes are restored resulting in an increase in SCFAs and lower levels of proinflammatory/unfavourable microbial metabolites [86]. Thus, these data indicate that meeting dietary fibre intake guidelines is likely to inhibit protein fermentation (proteolytic fermentation) and counteract any potential negative downstream effects.

It is acknowledged that a combination of factors including exercise, dietary habits and gut microbiota composition can influence the overall gastrointestinal function [56]. Nevertheless, given the prevalence of gastrointestinal symptoms in athletes [43, 87, 88], combined with elevated protein requirements, it is reasonable to recommended sufficient dietary fibre is ingested to maintain athlete microbiome diversity and corresponding gastrointestinal function [89, 90] (Table 3). Further research is required to determine whether fibre intake is associated with, or a direct cause of, observed health and performance benefits in athletes.

## Beta-Glucan

Beta-glucan is a water-soluble fibre derived from the cell walls of yeast, fungi, algae and oats. Beyond the established health benefits [91], beta-glucan ingestion may have direct relevance for athletes. For example, beta-glucan ingestion (~ 2 g per day) over a duration of 2–4 weeks has been reported to improve the parameters of strength [92] and endurance capacity [92]. The ingestion of beta-glucan has also been associated with reducing the inflammatory response after exercise [93], which may be relevant for athlete recovery. Therefore, although further research is required in exercise, it is reasonable to recommend that athletes include 2 g of beta-glucan (i.e. from oats and barley) within the total daily fibre intake.

## Personalised Response: One “Fibre” Size Does Not Fit All

Fibre ingestion is beneficial in individuals with normal microbial fermentative potential [40]. However, interventional studies have also shown that fibre fermentation has the potential to have detrimental effects in specific circumstances [95–96]. For example, individuals with reduced fibre-fermenting microbes and altered intestinal epithelial barrier permeability increased reactive oxygen species production and inflammation when ingesting fructooligosaccharides [95, 97]. Conversely, enhanced barrier integrity and reduced inflammatory response were observed when individuals with the corresponding fermenting microbes ingested the same fibre [96, 98].

Furthermore, overall microbial diversity does not change simply in response to increasing the dietary fibre intake (i.e. from 21.5 ± 8.0 g/day to 45.1 ± 10.7 g/day) [96]. In this study, analysis of blood proteomics allowed individuals to be divided into “high” or “low” inflammatory profiles (systemic cytokine concentration score). A low microbial diversity was characteristic of a high inflammatory response compared with the low inflammation group following the ingestion of the same high-fibre diet. Short-duration studies may not allow for the recruitment of new taxa to the microbiota (i.e. fibre-induced microbiota diversity may be a slower process requiring longer that 6 weeks). Nevertheless, in some individuals, the strategy to simply increase the intake of dietary fibre would not be appropriate to reverse any identified deficiencies of a “dysbiotic” gut microbiota. Thus, an individual gut microbiome assessment may allow individualised recommendations prior to a dietary fibre recommendation [56].

## Practical Application

Total daily fibre ingestion should be ≥ 30 g/day to achieve a fibre-derived health benefit in healthy adults [100–102]. However, this quantity of dietary fibre can be challenging to achieve as well as difficult to tolerate, especially in individuals lacking the aforementioned fibre-degrading species within their microbiome. Furthermore, antibiotics, non-steroidal anti-inflammatory drugs and sanitation, all commonly used by athletes, may cause substantial declines in gut microbial diversity, as well as a loss in fibre-degrading species [10, 103].

In the absence of strong mechanistic, clinical and epidemiological data, our opinion is to complete an initial evaluation of the daily fibre intake. Tolerance to dietary fibre can improve over time as the gastrointestinal motility and gut microbiota adapt to the quantity provided. Thus, athletes may first moderately raise the daily intake of fibre (i.e. structuring a ramp phase) while evaluating gastrointestinal symptoms [104, 105], as the adaptation rate is likely to differ between individuals (from 3 days to 2 weeks) [106, 107].

Sufficient volumes of fluid should be imbibed to accompany an increase in fibre intake. Drinking fluids helps speed fibre transit through the digestive system, whilst reducing the risk of constipation. This is particularly important for athletes who lose significant volumes of fluid during exercise as a consequence of thermoregulatory sweating [108].

**Fig. 3:**
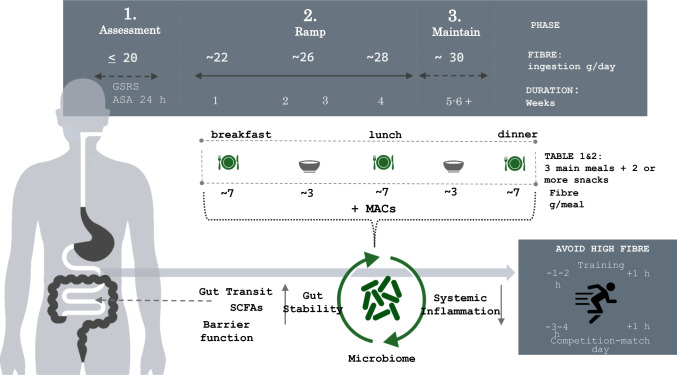
Guideline of increasing dietary fibre intake in athletes. Practical theoretical framework for athletes to achieve dietary fibre intakes. General fibre recommended daily amount quantities for men and women are used [96]. Top*:* a schematic aimed at achieving adequate dietary fibre ingestion. Following an initial evaluation of dietary fibre intake (ASA 24 h) and gastrointestinal symptoms (Gastrointestinal Symptom Rating Scale [GSRS]), a 4-week “ramp phase” period is recommended. Athletes should gradually increase the intake of fibres from ~ 20 to ~ 30 g with the aim of adding at least 10 g of fibre to their baseline consumption followed by a 2-week “maintenance phase”. Middle*:* a daily distribution of dietary fibre according to the main meals. Each main meal includes by ~ 7 g of fibre, while a snack throughout the day contains ~ 3 g. Daily adequate intakes of dietary fibres (microbiota accessible carbohydrates [MACs]) provide benefits for gastrointestinal health and gut microbiota composition and function (production of short-chain fatty acids [SCFAs]. Bottom: general recommendations for fibre management around exercise training and competition

Finally, constructing a diet with a sufficient dietary fibre is challenging given the large proportion of the day athletes dedicate to preparing for, participating in or recovering from exercise. As fibre-dense foods decrease gastric emptying and increase the risk of exacerbating exercise-associated gastrointestinal symptoms, it is intuitive to avoid them acutely around the exercise occasion [4]. However, to date, no studies have investigated optimal fibre intake strategies for athletes around daily, weekly or monthly training cycles or in competition. Instead, the recommended daily amount for fibre is aligned to that for the general population. In response, Fig. 3 provides a practical framework for athletes to achieve dietary fibre intakes.

## Conclusions

Dietary fibres play a crucial role in maintaining microbiome diversity and sustaining gastrointestinal function. Ingesting appropriate quantities of fibre in the daily diet may help athletes to maintain gastrointestinal health whilst meeting other nutrient intakes recommended to support training and exercise performance. Nevertheless, there are scenarios in which acute manipulation of fibre intake, either to reduce or increase intake, may be valuable in assisting the athlete to maintain gastrointestinal comfort during exercise or to contribute to body mass/composition goals. Establishing personalised guidelines for dietary fibre intake in athletes is warranted.
